# Helical intensity-modulated radiotherapy of the pelvic lymph nodes with a simultaneous integrated boost to the prostate - first results of the PLATIN 1 trial

**DOI:** 10.1186/s12885-015-1886-5

**Published:** 2015-11-07

**Authors:** Gregor Habl, Sonja Katayama, Matthias Uhl, Kerstin A. Kessel, Lutz Edler, Juergen Debus, Klaus Herfarth, Florian Sterzing

**Affiliations:** 1 Heidelberg Institute of Radiation Oncology (HIRO), Heidelberg, Germany; 2 Department of Radiation Oncology, Technische Universität München (TUM), Munich, Germany; 3 Department of Biostatistics, German Cancer Research Center, Heidelberg, Germany

**Keywords:** Prostate, Radiotherapy, Pelvic lymph nodes, IMRT, Tomotherapy, Antihormonal Treatment, Hypofractionation, Antihormonal therapy, Simultaneous integrated boost

## Abstract

**Background:**

Definitive, percutaneous irradiation of the prostate and the pelvic lymph nodes in high-risk prostate cancer is the alternative to prostatectomy plus lymphadenectomy. To date, the role of whole pelvis radiotherapy (WPRT) has not been clarified especially taking into consideration the benefits of high conformal IMRT (intensity modulated radiotherapy) of complex-shaped target volumes.

**Methods:**

From 2009 to 2012, 40 patients of high-risk prostate cancer with an increased risk of microscopic lymph node involvement were enrolled into this prospective phase II trial. Patients received at least two months of antihormonal treatment (AT) before radiotherapy continuing for at least 2 years. Helical IMRT (tomotherapy) of the pelvic lymph nodes (51.0 Gy) with a simultaneous integrated, moderate hypofractionated boost (single dose of 2.25 Gy) to the prostate (76.5 Gy) was performed in 34 fractions. PSA levels, prostate-related symptoms and quality of life were assessed at regular intervals for 24 months.

**Results:**

Of the 40 patients enrolled, 38 finished the treatment as planned. Overall acute toxicity rates were low and no acute grade 3 or 4 gastrointestinal (GI) and genitourinary (GU) toxicity occurred. 21.6 % of patients experienced acute grade 2 but no late grade ≥2 GI toxicity. Regarding GU side effects, results showed 48.6 % acute grade 2 and 6.4 % late grade 2 toxicity. After a median observation time of 23.4 months the PLATIN 1 trial can be considered as sufficiently safe meeting the prospectively defined aims of the trial. With 34/37 patients free of a PSA recurrence it shows promising efficacy.

**Conclusion:**

Tomotherapy of the pelvic lymph nodes with a simultaneous integrated boost to the prostate can be performed safely and without excessive toxicity. The combined irradiation of both prostate and pelvic lymph nodes seems to be as well tolerated as the irradiation of the prostate alone.

**Trial registration:**

Trial Numbers: ARO 2009–05, ClinicalTrials.gov: NCT01903408.

## Background

Percutaneous irradiation of locally advanced prostate cancer is the alternative therapy to radical prostatectomy (RP). A direct and valid comparison between radiation therapy (RT) and RP is not possible on the basis of existing studies due to the lack of prospective clinical trials [1–3]. When comparing retrospectively the clinical outcome of external beam radiotherapy (EBRT) with case series for RP, similar rates of the five-year (5y) biochemical progression free survival (bPFS) and the 5y- and 10y-disease-specific survival (DSS) were observed for EBRT, while the 10y-bPFS and the 10y-overall survival (OS) were slightly higher in favor of RP. However, this weak evidence does not allow a prioritization of one of the two treatments for locally advanced prostate cancer.

The addition of pelvic lymph nodes to the radiation field raised concerns regarding possible increased side effects. Acute gastrointestinal (GI) and genitourinary (GU) toxicities were more frequently reported in patients treated with WPRT [4, 5]. Late complications were also more frequently seen in the whole pelvis (WP)RT group compared to postoperative radiotherapy (PORT) [6–8]. IMRT can reduce acute and late toxicities using smaller irradiated volumes of bladder, small bowels and rectum [9, 10]. Moderately hypofractionated RT has become routine over the years due to area-wide implementation of image guided (IG)RT and better knowledge of tumor radiation biology [11–13]. Hence, in the present study we combined both, advanced IMRT/IGRT techniques for a better tolerability, and a moderate hypofractionated simultaneous integrated boost (SIB) to the prostate for a probably higher biological effectiveness.

The PLATIN (Prostate and Lymph Node Irradiation with Integrated-Boost-IMRT after NHT) phase II trial evaluates an optimized WPRT in patients with locally advanced prostate cancer. Due to image guided IMRT even inhomogeneous dose distributions can be applied accurately. Initiated in 2009, the study was designed to prospectively investigate safety and feasibility of five prostate and lymph node irradiation concepts, each enrolling *n* = 40 patients. Pelvic lymph nodes are simultaneously irradiated with an integrated boost to either the prostate (PLATIN 1), the prostate and macroscopic lymph nodes (PLATIN 2), the prostate bed (PLATIN 3), the prostate bed and macroscopic lymph nodes (PLATIN 4), or to macroscopic lymph nodes in patients with prior PBRT (PLATIN 5). Secondary objectives were a detailed characterization of the toxicity profiles, and the evaluation of quality of life during treatment. Results of the PLATIN 3 arm were published by Katayama et al. [14].

In this article we report on safety and efficacy data applying IMRT treatment of the pelvic lymph nodes with a SIB to the prostate (PLATIN 1).

## Methods

From May 2009 to December 2012, 40 patients were enrolled prospectively in the PLATIN 1 trial. Eligibility criteria were, among others, a histological proven prostate carcinoma without lymph node metastases but with an estimated risk of lymph node involvement >20 % according to the Roach formula [15]. In case of a diagnostic lymphadenectomy, a minimum number of ten lymph nodes had to be surgically removed.

Before trial initiation, ethical consent was obtained from the ethics committee of the University of Heidelberg (permit S-034/2009). All patients gave written informed consent before trial enrollment. All reported data were conducted in accordance with the Helsinki Declaration and with national guidelines. Patients received at least 2 months of neoadjuvant AT (bicalutamide or LHRH analogue). With good tolerability AT was continued for at least 2 years after irradiation.

For treatment planning, CT scans with 3 mm slice thickness at full bladder and empty rectum were performed. PTV-P (planning target volume - prostate) covered the prostate (CTV-P) + 6 mm including the seminal vesicles. PTV-L (planning target volume - lymph nodes) included the obturatory, internal and external iliac, common iliac and presacral (down to S3) lymph nodes with a 5 mm margin [16]. Pararectal lymph nodes were not included in the PTV-L. Inverse treatment planning was performed using the Tomotherapy® treatment planning software (Accuray, USA). A total dose of 51.0 Gy was prescribed to 95 % of PTV-L with a SIB of 76.5 Gy to 95 % of PTV-P in 34 fractions. The dose prescription to the lymph nodes of 51 Gy in 1.5 Gy fractions is biologically equivalent to 43.7 Gy, assuming a α/β of 1.5 Gy for prostate cancer; and 48.2 Gy, assuming a α/β of 7 Gy for small bowel. Treatment was performed with full bladder and empty rectum under daily IGRT.

Prostate-specific symptoms and treatment toxicity, using the criteria of the NCI CTC AE version 3.0, were recorded before treatment, weekly during treatment, at the end of treatment, and at 2.5, 6, 12, 18 and 24 months follow-up. For calculation of toxicity rates, only patients with available data at the respective time points were considered. Cumulative GI toxicity was defined as the cumulative incidence of diarrhea, enteritis and proctitis. To facilitate comparison with other publications, only cystitis was included in the calculation of cumulative GU toxicity, as most scoring systems do not include incontinence and erectile dysfunction. Nevertheless, incontinence and erectile dysfunction were recorded.

Quality of life was assessed using the EORTC QLQ-C30 questionnaire before treatment and during follow-up after 6, 12 and 24 months.

PSA levels were measured before radiotherapy and then every 3 months afterwards, starting from week 10. Biochemical failure was established according to the Phoenix criteria [17].

A primary endpoint, the safe treatment application rate (STR) was chosen. STR was defined as the proportion of patients receiving treatment as planned and without grade 3–4 toxicity and calculated as the ratio of the number of patients fulfilling this criteria divided by the size of the Intention-to-treat (ITT) population. The ITT population consisted of all patients giving informed consent, fulfilling the inclusion and exclusion criteria and receiving planned treatment for a minimum of 4 weeks after initiation. Based on a one-stage phase II type design, STR of 80 % (null-hypothesis SDR ≤80 %) was tested against the alternative of being at least as large as 95 % in a one-stage phase-II type design using the exact binomial test at the significance level of 0.1 % with a power of 90 %. The null hypothesis would be rejected when SDR would be at least 87.7 %.

## Results

### Patient characteristics

Among all 40 patients (identical with the ITT population), median follow-up was 23.4 months (range: 2.8 – 31.7 months). Median age at inclusion was 70 years (range: 51 – 75 years); all patients were high-risk according to the D‘Amico risk categories [18]. One patient underwent laparoscopic resection of lymph nodes but rejected prostatectomy. Indication for WPRT was seen by reason of lymph node affection (pN+) without remaining macroscopic lymph node metastasis in situ. All other patients had an estimated risk of lymph node involvement >20 % according to the Roach formula. Both LHRH and antiandrogen therapy were permitted as antihormonal therapies.

Twenty-seven patients received LHRH analogue therapy, seven patients received bicalutamide and six patients both (complete androgen deprivation). Most of the patients still had antihormonal therapy prescribed by their urologist as they were seen in the Department of Radiooncology. Radiotherapy was performed as definitive treatment in 38 patients. Two patients had PSA elevation during NHT and were thus excluded from the study. One patient died 7 months after radiotherapy diagnosed with a metastasized esophageal cancer. For further patient characteristics see Table 1.

**Table 1:** Patient characteristics of the included patients (*n* = 40): T- and N-stage, initial PSA (ng(ml) and Gleason-Score

T stage	
T1c	22
T2a	2
T2b	1
T2c	4
T3a	4
T3b	6
T4a	1
N stage	
N0	39
N1	1
PSA (ng/ml)	
<10	12
10–20	10
20–30	9
30–40	2
40–50	4
>50	3
Gleason-Score	
7(3 + 4)	9
7(4 + 3)	11
8(4 + 4)	15
8(5 + 3)	1
9(4 + 5)	3
10(5 + 5)	1

### Treatment characteristics

Average beam on time of the 38 evaluated radiation plans was 477 ± 78 s. The intended target coverage could be met. 95 % of the PTV-P received 75 ± 2.5 Gy (median dose: 78.5 ± 0.5 Gy) and 95 % of the PTV-L received 50.7 ± 0.5 Gy (median dose: 53.8 ± 1.1 Gy).

Plan quality in terms of organ at risk sparing is shown in Table 2. The anterior rectal wall received a maximum dose of 72.8 ± 1.3 Gy. The rectum received doses ≥60 Gy (9.5 %) and ≥70 Gy (1.6 %). Dose to the small bowel could be kept low in the segmented parts with 9.4 % of the small bowel exposed to ≥40 Gy and a maximum dose of 52.7 Gy. Most of the bladder could be spared from high dose exposure with 6.3 % of the bladder receiving ≥70 Gy.

**Table 2:** Average dose exposure to the rectum, small bowel and bladder

Rectum	
Maximum anterior rectal wall	73.7 Gy ± 2.1 Gy
V_40 Gy_	39.1 % ± 10.5 %/38 ml ± 10 ml
V_60 Gy_	9.3 % ± 3.8 %/9 ml ± 4 ml
V_70 Gy_	1.6 % ± 1.7 %/2 ml ± 2 ml
Small bowel	
Maximum	52.8 Gy ± 1.2 Gy
V_20 Gy_	42.9 % ± 19.0 %/664 ml ± 293 ml
V_40 Gy_	9.3 % ± 6.1 %/143 ml ± 94 ml
Bladder	
V_40 Gy_	40.6 % ± 12.4 %/142 ml ± 44 ml
V_60 Gy_	12.0 % ± 6.5 %/42 ml ± 23 ml
V_70 Gy_	6.3 % ± 4.1 %/22 ml ± 15 ml

*V*_*20 Gy*_*, V*_*40 Gy*_*, V*_*60 Gy*_*, V*_*70 Gy*_ volume of the respective organ at risk receiving ≥ 20 Gy, ≥ 40 Gy, ≥ 60 Gy and ≥ 70 Gy

### Treatment safety

After a median observation time of 23.4 months one patient out of 38 died seven months after irradiation suffering from metastasized esophageal cancer. No patient showed acute toxicity ≥grade 3. We defined acute toxicity as side effects within 6 months after therapy. Therefore, at the time of evaluation the PLATIN 1 trial met the prospectively defined statistical criteria of a successful treatment with an SDR of at least 87.7 %.

### Gastrointestinal toxicity

Cumulative incidence of acute GI toxicity was 56.8 % (grade 1) and 21.6 % (grade 2). No acute grade 3 or 4 GI toxicity occurred. During treatment, patients suffered from diarrhea in 18.9 % (grade 1) and 2.7 % (grade 2). Proctitis was reported in 13.5 % (grade 1) and 5.4 % (grade 2) of all cases. Enteritis grade 1 occurred in 15.4 % and grade 2 in 2.7 %.

Cumulative late GI toxicity was 6.1 % (grade 1). No patient suffered from late enteritis of any grade. Only one patient experienced late proctitis grade 1 at 12 months of follow-up, and one late diarrhea grade 1 at 18 months was observed (see Table 3).

**Table 3:** Acute and late gastrointestinal toxicity

	Grade 1	Grade 2	Grade 3	Grade 4
Diarrhea				
End of RT	18.9 %	2.7 %	–	–
13 weeks	13.9 %	–	–	–
6 months	5.9 %	2.9 %	–	–
12 months	–	–	–	–
18 months	3.2 %	–	–	–
24 months	–	–	–	–
Enteritis				
End of RT	13.5 %	2.7 %	–	–
13 weeks	2.8 %	–	–	–
6 months	2.9 %	2.9 %	–	–
12 months	–	–	–	–
18 months	–	–	–	–
24 months	–	–	–	–
Proctitis				
End of RT	13.5 %	5.4 %	–	–
13 weeks	2.8 %	–	–	–
6 months	–	–	–	–
12 months	2.9 %	–	–	–
18 months	–	–	–	–
24 months	–	–	–	–

*RT* radiotherapy

### Genitourinary toxicity

The incidence of acute GU toxicity is comparable to other published data with 78.4 % (grade 1) and 48.6 % (grade 2). Cumulative incidence of late GU toxicity was 12.3 % (grade 1) and 6.4 % (grade 2). No patient developed acute or late GU toxicity grade 3/4.

Acute cystitis was reported in 35.1 % (grade 1) and 18.9 % (grade 2) of all patients (see Table 4). Two patients reported stress incontinence grade 1 (occasional, no pads necessary) at the beginning of radiation therapy, which regressed about 12 months thereafter. Four other patients reported on stress incontinence grade 1 in one follow-up. In all cases, the symptoms resolved until the following visit.

**Table 4:** Acute and late cystitis

Cystitis	Grade 1	Grade 2	Grade 3	Grade 4
End of RT	35.1 %	18.9 %	–	–
13 weeks	8.3 %	5.6 %	–	–
6 months	2.9 %	2.9 %	–	–
12 months	9.1 %	3.0 %	–	–
18 months	3.2 %	3.2 %	–	–
24 months	–	–	–	–

*RT* radiotherapy

One patient required urinary catheterization (for 8 days) during follow-up (6 days after the end of radiotherapy) due to urinary retention. As no other intervention was required, it was rated as grade 2 toxicity. For all other patients the urinary flow has hardly changed during and after therapy. At 24 months of follow-up, all patients were catheter-free.

Urge incontinence increased after treatment within the first 6 months from 8.1 to 20.5 % (grade 1) and from 2.7 to 2.9 % (grade 2). At 24 months, urge incontinence decreased to 7.4 % (grade 1) and 0 % (grade 2).

Within the study, we also evaluated the incidence of adverse effects on libido and erectile dysfunction. However, since hormonal therapy is running within the first 2 years, toxicity analysis is reasonable only after discontinuation of AHT.

### Quality of life

Overall health as assessed by the “Global Health Score” of the EORTC QLQ-C30 questionnaire remained almost unchanged at 6, 12 and 24 months follow-up compared to baseline (see Table 5). Scores were on a similar level as the EORTC reference value of prostate cancer patients over all disease stages.

**Table 5:** Evaluation of the scores of EORTC QLQ-C30 v. 3.0

	Before RT	Month 6	Month 12	Month 24
Global health status/QoL	67 %	67 %	67 %	83 %
Physical functioning	93 %	87 %	87 %	87 %
Role functioning	100 %	100 %	92 %	83 %
Emotional functioning	83 %	83 %	92 %	83 %
Cognitive functioning	83 %	83 %	83 %	100 %
Social functioning	100 %	100 %	100 %	100 %
Fatigue	11 %	22 %	22 %	22 %
Nausea and vomiting	0 %	0 %	0 %	0 %
Pain	0 %	0 %	0 %	0 %
Dyspnea	0 %	33 %	33 %	0 %
Insomnia	0 %	0 %	0 %	0 %
Appetite loss	0 %	0 %	0 %	0 %
Constipation	0 %	0 %	0 %	0 %
Diarrhea	0 %	0 %	0 %	0 %
Financial difficulties	0 %	0 %	0 %	0 %

### Biochemical control and survival

**Fig. 1:**
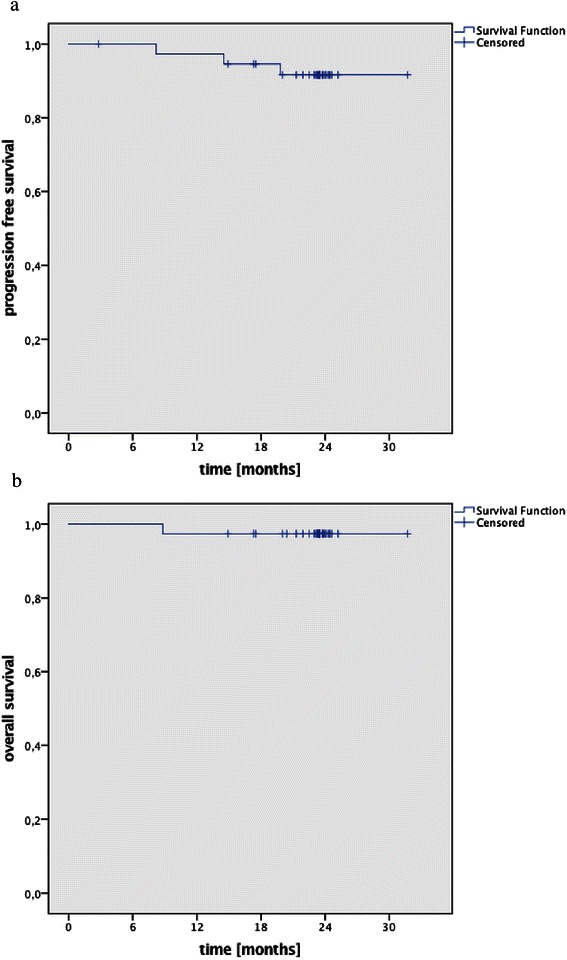
Actuarial biochemical progression free survival (**a**) and overall survival (**b**)

During follow-up, three patients experienced PSA recurrences. All three had discontinued AT after radiotherapy resulting in an actuarial biochemical progression free survival of 91.9 % after 2 years (see Fig. 1a). In one patient, PSA recurrence coincided with the diagnosis of bone metastases; for the other two patients, the localization of recurrence could not be determined. Ten patients quit AT after completion of radiotherapy due to reported intolerance and side effects. At the time of analysis, 15 patients still received AT. Average duration of AT was 13.6 months for the 38 patients remaining in the trial after the end of radiotherapy. At a median of 24 months follow-up, 37 patients were alive resulting in an actuarial overall survival (OS) of 97.3 % (see Fig. 1b).

## Discussion

The procedure of EBRT for prostate cancer has undergone many changes in the past decade. Recently, dose escalation, hypofractionation and the use of IMRT and IGRT became a standard method. Hence, outcome data are slowly emerging. The necessity of prophylactic radiotherapy of the pelvic lymph nodes remains controversial. Many radiation oncology centers electively treat the pelvic lymph nodes because of publications on surgical lymph node sampling and nanoparticle-enhanced MRI studies that revealed a high proportion of occult lymph node metastases [19, 20]. In contrast, other centers avoid WPRT because of concerns about excessive toxicity. For more clarity, the effect of WPRT was tested in large prospective trials. Unfortunately, the long-term PFS showed no significant differences between WPRT and PORT [21–23]. However, these trials were designed before the dose-escalation era [6, 7, 24, 25]. With a dose-escalation up to 75.6 Gy to the prostate, Aizer et al. could prove the superiority of WPRT [8]. Especially in high-risk situations a benefit with reduced rates of failure were seen in patients treated with doses higher than 75 Gy [26, 27].

The present phase II study shows good tolerability of IMRT-based treatment of the prostate and pelvic lymph nodes. We revealed a reduced incidence of toxicities compared to conventionally fractionated schemes. The RTOG 94–13 trial showed late grade ≥3 GI and GU toxicities at 5 years of 3.0 and 4.3 %, respectively, using conventionally fractionation of 1.8 Gy (50.4 Gy WPRT + 19.8 Gy boost to the prostate bed). The GETUG-01 trial applied doses to the prostate and whole pelvis of 66 – 72 Gy à 1.8 – 2.25 Gy and 45–46.8 Gy à 1.8 Gy. Here, 37.7 % of patients developed late grade ≥2 GU toxicity and 31.7 % developed late grade ≥2 GI toxicity. In our trial, the observed rates of late toxicity compare favorably to the mentioned studies with no grade ≥2 GI toxicity, and 6.4 % grade 2 and no grade 3 or 4 GU toxicity. On the one hand, the lower rates of side effects could be attributed to the modern IMRT/IGRT technique; on the other hand, the moderate hypofractionation could have an important impact: the α/β ratio of prostate cancer is supposed to be lower than the surrounding healthy tissue of rectum and bladder resulting in a probably lower rate of late side effects.

In our trial, patients received at least 2 months of neoadjuvant AT. With good tolerability AT was continued for at least 2 years after irradiation. The addition of antihormonal treatment (AT) in patients with clinically localized intermediate or high-risk prostate cancer showed superior results (bPFS, DSS, OS) in a number of prospective randomized trials [28–31]. The question of the right timing of adjuvant hormonal treatment (AHT) is still under debate and lasts from 4 months in the RTOG 86–10 trial to 3 years in the EORTC trial [32–35]. Bolla et al. demonstrated a significantly lower overall mortality in patients under neoadjuvant hormonal treatment (NHT) plus 3 years AHT compared to 6 months AHT [36]. Most randomized phase III trials used short-term NHT in combination with EBRT and showed an improvement either in absence of PSA failure or OS [33, 37, 38]. One of the possible effects of NHT is the immune modulation of AT resulting in T-cell infiltration of the prostate, which can increase apoptosis [39]. Most studies using long-term AHT for high-risk patients added whole pelvis radiotherapy (WPRT) [30, 32, 40]. The RTOG 92–02 trial proved that NHT combined with long-term AHT is superior to NHT for locally advanced/high-risk patients [40]. While it is still unclear whether an additional WPRT is beneficial compared to prostate irradiation alone [21, 22, 41, 42], the mentioned studies also showed no significant improvement of relevant clinical endpoints (OS, bPFS, DSS). The RTOG 94–13 trial planned to evaluate the timing of AT. The role of WPRT became rather complex as the authors found an interaction between field size and timing of AT. No benefit could be found in the trial testing WPRT vs. prostate only RT (PORT) in combination with AHT vs. NHT [21]. However, the results suggest that if a patient chooses NHT, WPRT appears beneficial compared to PORT. The risk of high-grade (> grade 3) GI toxicity is potentially higher under additional WPRT and AT compared to PORT.

The evaluation of erectile function and libido based on patient-reported data is prone to reporting bias. Nevertheless, we documented the incidence of adverse effects on libido and erectile dysfunction as well. At this stage evaluation is too early, as for most patients AT is still running within the first 2 years of follow-up. Toxicity analysis is only reasonable if AT is completed.

Our study has shown both safety and efficacy using helical tomotherapy. At the point of analysis, 34 of 37 patients were free from PSA recurrence. However, it is important to note the limitations of our first analysis. The patient number is relatively small. For a reliable evaluation of efficacy, a median follow-up of 23.4 months is rather short. A longer follow-up period is needed to detect differences especially in late toxicity. Also, it will provide more significant reports on biochemical control.

## Conclusion

While the role of WPRT of prostate cancer remains to be fully explored, we could demonstrate in the prospective PLATIN 1 trial that prophylactic radiotherapy of the pelvic lymph nodes with a SIB to the prostate can be performed without excessive toxicity. The combined irradiation of both prostate and pelvic lymph nodes seems to be as well tolerated as the irradiation of the prostate alone.
